# Particulate emissions from diesel engines: correlation between engine technology and emissions

**DOI:** 10.1186/1745-6673-9-6

**Published:** 2014-03-07

**Authors:** Michael Fiebig, Andreas Wiartalla, Bastian Holderbaum, Sebastian Kiesow

**Affiliations:** 1 Institution: FEV GmbH, Neuenhofstraße 181, 52078 Aachen, Germany

**Keywords:** Diesel engine technology, Diesel engine emissions, Particulate mass emission, Particle number emission, Technological progress

## Abstract

In the last 30 years, diesel engines have made rapid progress to increased efficiency, environmental protection and comfort for both light- and heavy-duty applications. The technical developments include all issues from fuel to combustion process to exhaust gas aftertreatment. This paper provides a comprehensive summary of the available literature regarding technical developments and their impact on the reduction of pollutant emission. This includes emission legislation, fuel quality, diesel engine- and exhaust gas aftertreatment technologies, as well as particulate composition, with a focus on the mass-related particulate emission of on-road vehicle applications. Diesel engine technologies representative of real-world on-road applications will be highlighted.

Internal engine modifications now make it possible to minimize particulate and nitrogen oxide emissions with nearly no reduction in power. Among these modifications are cooled exhaust gas recirculation, optimized injections systems, adapted charging systems and optimized combustion processes with high turbulence. With introduction and optimization of exhaust gas aftertreatment systems, such as the diesel oxidation catalyst and the diesel particulate trap, as well as NOx-reduction systems, pollutant emissions have been significantly decreased. Today, sulfur poisoning of diesel oxidation catalysts is no longer considered a problem due to the low-sulfur fuel used in Europe. In the future, there will be an increased use of bio-fuels, which generally have a positive impact on the particulate emissions and do not increase the particle number emissions.

Since the introduction of the EU emissions legislation, all emission limits have been reduced by over 90%. Further steps can be expected in the future. Retrospectively, the particulate emissions of modern diesel engines with respect to quality and quantity cannot be compared with those of older engines. Internal engine modifications lead to a clear reduction of the particulate emissions without a negative impact on the particulate-size distribution towards smaller particles. The residual particles can be trapped in a diesel particulate trap independent of their size or the engine operating mode. The usage of a wall-flow diesel particulate filter leads to an extreme reduction of the emitted particulate mass and number, approaching 100%. A reduced particulate mass emission is always connected to a reduced particle number emission.

## Introduction

From a technical perspective, diesel engines are caught in an area of conflict between a wide variety of requirements ranging from maximum customer benefit, minimum fuel consumption, to minimum emissions. While CO_2_ output has only recently been regulated, statutory emissions limits have been in place since the 1970s. In the beginning of the 1990s, the “Euro” emission standard was introduced, where limits continue to become more stringent in individual emission levels. The currently valid legislation level Euro 5 will be replaced by Euro 6 in 2014. To comply with the legislative limits, or to comply with the future limits prior to the deadlines and to simultaneously meet all customer requirements, systematic further development of diesel engines is necessary. While the basic principle has not changed, the process cycle technology of advanced engines has been optimized considerably. In addition to further developing the engines, exhaust aftertreatment systems were introduced in order to additionally lower engine emissions.

The objective of this paper is to provide the most comprehensive summary possible of the literature available on the technical further development and its effect on the reduction of emissions, in particular the reduction of particulate emissions. The emphasis will be on showing the effects of further diesel engine developments on mass-related particulate emissions with the focus on vehicle applications for road use; industrial engines and marine applications will not be considered. Where it makes sense, the effects will be shown separately for passenger cars and commercial vehicles (CVs). The emphasis will be on comparative studies of different technology stages under comparable boundary conditions. In view of the fact that diesel engines – in particular in the passenger car sector – can look back on a long and successful history especially in Europe and Germany, a large portion of the literature shown here comes from the German and European region. This paper also aims to shed light on diesel engine technology, which is representative for drive assemblies actually used in road traffic. This is why publications that refer to actual series production engines are particularly relevant.

## Diesel engine operation

The diesel engine is an internal combustion engine that generates mechanical work from the chemically bound energy of fuel by means of combustion. To do this, a 4-stroke cycle is typically used, where fresh air is taken in (cycle 1) and compressed in the combustion chamber (cycle 2). The fuel is injected near the end of the compression and the inhomogeneous fuel/air mixture self-ignites at a high compression temperature. Due to the expansion of the gas in the combustion chamber, the piston is moved and output is generated (cycle 3); finally, the burnt mixture is discharged (cycle 4). The engine load output is determined by the injected fuel mass, whereby the mixing ratio of fuel and air in the combustion chamber changes (mixture quality). In addition to the products of complete combustion (carbon dioxide (CO_2_) and water (H_2_O)), this type of combustion also generates a few undesirable pollutants, mainly carbon monoxide (CO), unburnt hydrocarbons (HC), nitrogen oxides (NO_x_), and particles (PM).

## Emissions legislation and fuel grade

**Figure 1:**
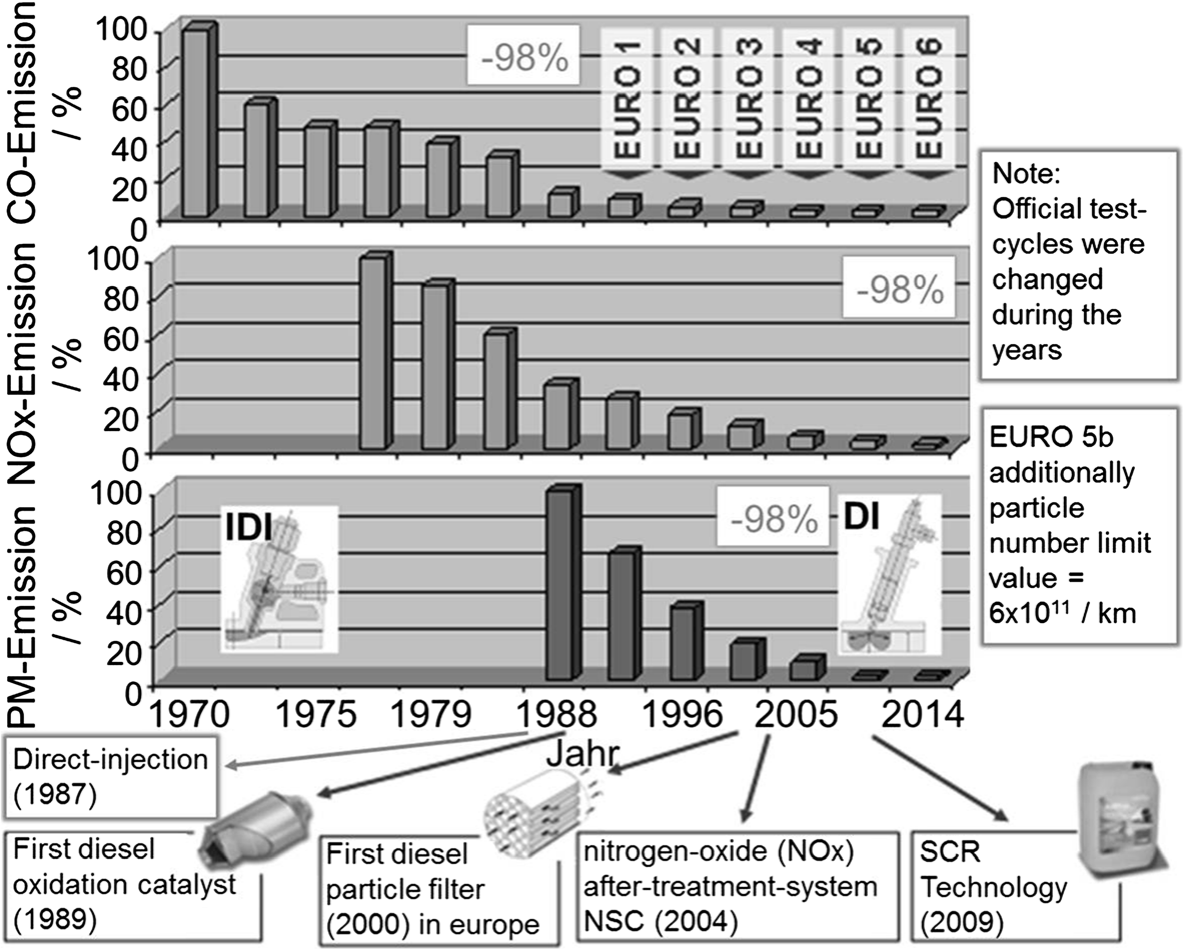
**Development of european emission limit values for diesel passenger cars and introduction of diesel exhaust gas after treatment technologies **[1]**.**

These pollutants have been limited for passenger cars and/or commercial vehicles since the introduction of the emissions legislation with emission standard Euro 1 in Europe. The development of the European emission limits for passenger cars is shown in Figure 1. There has been a significant reduction by approximately 98% in all emissions from Euro 1 to Euro 6. With the Euro 5b standard, a limit value for particle number (PN) was additionally introduced.

**Figure 2:**
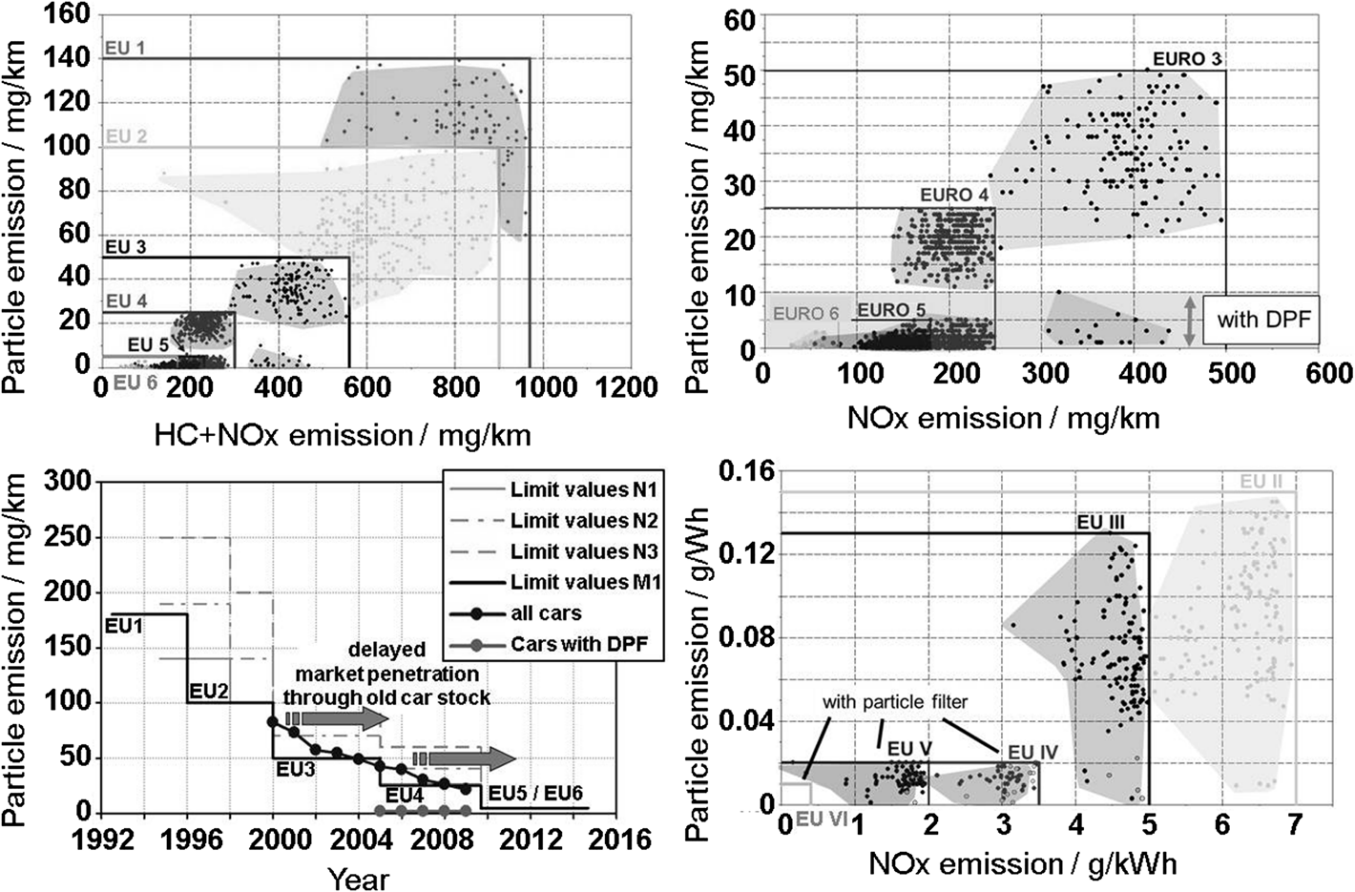
**KBA emission limits values.** Limit values for passenger cars (upper left). Detailed view of EURO 3 to 6 norm for passenger cars (upper right). Limit values for commercial vehicles (lower right). Market penetration of new emission limit levels for passenger cars [2].

**Figure 3:**
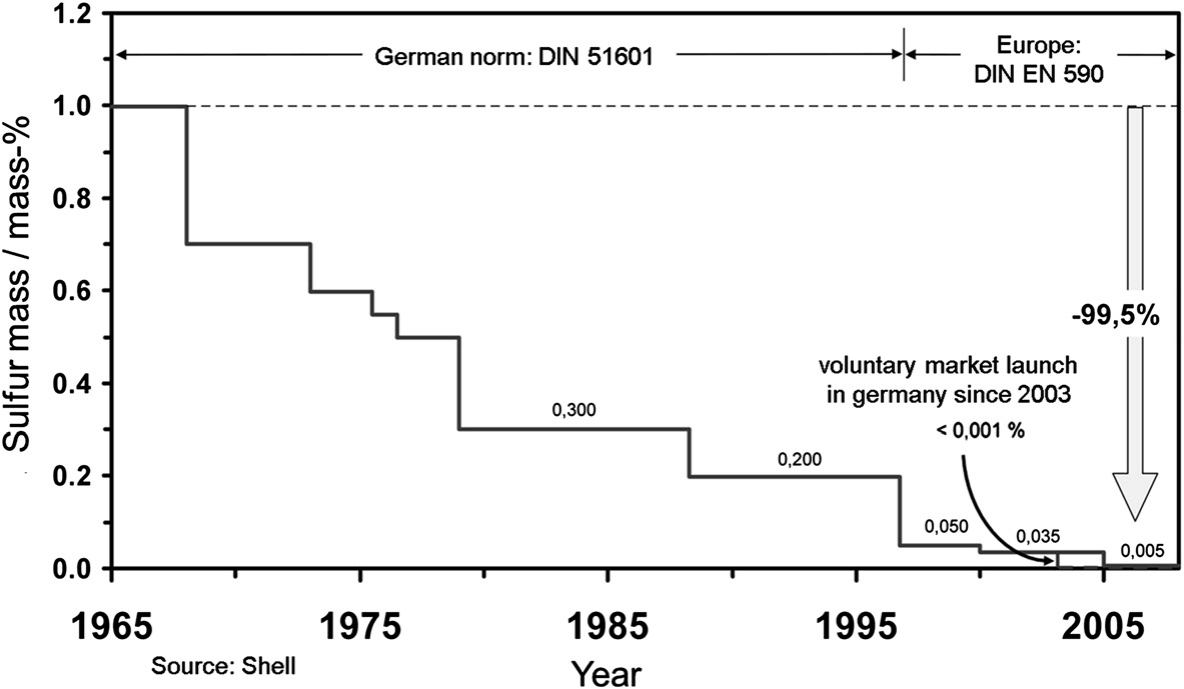
Development of sulfur amount in diesel fuel since 1965.

Comparing the values of registered vehicles based on the exhaust type values published by the KBA [2] in terms of the development from Euro 1 to Euro 6, we can see that the values of registered vehicles have been clearly below the limits in some cases, which is true especially for particulate emissions of vehicles with diesel particulate filters (DPF) in emission standard Euro 3 and Euro 4 (Figure 2 above). Starting with Euro 5, the lowest particulate levels have been universally achieved by using DPFs. Euro 6 certified passenger cars are already available today on the market. The figure (on the lower left) additionally shows that a fast market penetration of new emission standards has been reached by new registered vehicles after these standards were introduced. Euro VI certified commercial vehicles have only been available on the market for a short period of time (Emission standards for commercial vehicles highlighted by a roman numeral). The development of the emission limits and KBA registration numbers showed a similarly fast and significant reduction in all emissions for commercial vehicles (on the lower right), while the values were far below the limits in some cases. Euro IV and Euro V concepts have been used in series production both with (open symbols) as well as without DPF (closed symbols), since the majority of commercial vehicle manufacturers has pursued in-engine particle reduction and a reduction in NO_x_ emissions using the SCR technology for Euro V because of the associated fuel consumption benefits. Only a few manufacturers have used in-engine NO_x_ reduction and a DPF. With Euro VI, a limit for the particles concentration will be introduced additionally for commercial vehicles, so that the universal introduction of the DPF can also be expected in commercial vehicle engines [3-5]. For the registration, the emission limits were verified in defined test cycles that differ world-wide. In passenger cars, the vehicle had been certified in the “New European Driving Cycle” (NEDC) on an exhaust gas roller dynamometer up until now; in the commercial vehicle sector, the engine was being certified on an engine test bed due to the high number of variants. Up to emission standard Euro II, only a stationary emission test (ECE R-49) used to be required for commercial vehicles in Europe. With the introduction of Euro III, it was replaced by a new stationary test (ESC), and a transient test (ETC.) as well as a smoke test (ELR) were added. With the introduction of the “Worldwide Harmonized Test Cycle” (WHTC) for commercial vehicle engines, an important step has been taken in the direction of world-wide harmonization; there were corresponding suggestions in the passenger car area. 2 new test cycles – WHSC (Worldwide Harmonized Stationary Cycle) and WHTC (Worldwide Harmonized Transient Cycle) – were used here. Due to an additional cold start test as well as the holding time prior to the warm test, these placed additional demands on emission reduction with their low exhaust temperature levels across the entire cycle. In addition to the new test cycles, there was also a so-called NTE (“Not-to-Exceed”) range, where the limit value was not to be exceeded by more than 50% at any engine operating point. Since the introduction of Euro 3 in the passenger car area and/or Euro IV in the commercial vehicle area, the emission levels not only had to be verified for new vehicles and/or new engines, but the stability of the emissions over the operating time also had to be verified. The current verification period for passenger cars is 160,000 km and up to 700,000 km for commercial vehicles [6]. Furthermore, all vehicles must be equipped with an on-board diagnosis system (OBD) starting with these emission standards. It monitors the function of the emission-relevant components, detecting a failure or defect of an emission-relevant component and showing it to the driver. The purpose is to prevent emission levels from being exceeded in the field.The emissions legislation is closely linked to the fuel grade provisions, since lowest emission limits and a wide range of new technologies are only possible in combination with the corresponding fuel grade. The concentration limit for sulfur in fuel that still used to be approximately 1% in 1965, was reduced to 0.2% in 1993 with the introduction of the Europe-wide regulation for diesel quality (DIN EN 590). Since then, the sulfur content has been further reduced by more than 99.5% to a current limit of 10 ppm (0.001%) (Figure 3).

## Particulate measurement technology

According to the EU directive from 1970, the particles found in the exhaust of internal combustion engines are defined as exhaust gas components, which are filtered at a temperature of max. 325 K (52°C) in the diluted exhaust using hydrophobic filters that are inert towards the exhaust gas components [7]. These essentially include carbon (soot), HCs with a higher boiling point that are partially adhering to the soot, and sulfates. Corrosion products from the engine and combustion products resulting from additives in the engine oil [8] also make up a small portion of the particulate emissions. The emitted particles differ with regard to their chemical composition, density, form, and electric charge. This makes a simple classification difficult, which is why a standardized equivalent diameter has become the typically used method. It corresponds to the diameter of a spherical particle, which, in a certain experiment [9], behaves like the particle to be described. A sphere with the same density and the same sedimentation velocity is referred to as Stokes equivalent sphere. The aerodynamic equivalent sphere has the same sedimentation velocity, but a normalized density of 1,000 kg/m^3^.

In the particulate measurement technology employed during the development of internal combustion engines and exhaust aftertreatment systems, a broad range of different systems were used. The particles were classified using different mechanisms with regard to their physical properties, which makes it more difficult to compare the obtained results. The procedures listed in Table 1 are commonly used [1,9-15]. Particularly problematic is the differentiation between solid and liquid components. The above definition of particles leads to a blurred boundary between these components, making a clear allocation between particles based on this definition and the term “soot” impossible. In order to measure solid particles only, it is possible to remove the volatile components for example by means of thermodesorption or two stage dilution [16]. To make the determined emission levels comparable and to reduce measurement errors, the measurements are regulated in details in legal provisions [17]. For the certification of vehicles and engines in terms of the particulate mass, the gravimetric analysis method is used. For passenger cars, the full flow dilution method is used [18], and for commercial vehicles, the partial flow method is used in addition. The measurement of emitted particles (mass, number) represents a big challenge, since the measured values do not only greatly depend on the measurement principle, but also on the sampling method. At the same time, the particulate limits are already very low today, making it necessary to verify the corresponding low particle numbers using measuring technology. In some cases, the concentrations in the exhaust to be recorded are considerably lower than the particle numbers in the ambient air (see also chapter “Particulate reduction systems”). These two factors result in high measurement inaccuracies, and a high amount of technical effort is necessary to obtain reliable measurement results.

**Table 1:** Particle measurement

**Method**	**Measured variable**	**Measured substance**
Gravimetry	Particulate mass	Particles as per definition^1)^
Opacity	Light absorption coefficient	Soot
stray light	Particle number	Solid particle with suitable conditioning^2)^
Impaction	Particle number and distribution in terms of aerodynamic diameter	Solid particle with suitable conditioning^2)^
Differential analysis of mobility	Particle number and distribution in terms of mobility diameter	Solid particle with suitable conditioning^2)^

^1)^Exhaust gas components that are filtered at a maximum temperature of 52°C after dilution of the exhaust gases with filtered air in a hydrophobic filter material with inert properties against the exhaust gas components are defined as particles (EU 1970).

^2)^Removal of volatile components through thermodesorption or two-stage dilution (heated/unheated).

## Progress in diesel engine technology

The technology used in diesel engines has been continuously further developed over the past 40 years, resulting in a wide range of effects on emissions and consumption.

### In-engine measures to reduce emissions

#### Fuel injection technology

Depending on the design of the injection system and the load, the injection pressure ranges from 150 to up to 2,200 bar. At full load, the injection has a crank angle interval from 15 to 50 °CA depending on the combustion system. Originally, indirect injection into a divided combustion chamber (pre-combustion or swirl chamber) which was connected to the main combustion chamber was used for mixture formation. This method was replaced by direct injection into the main combustion chamber [19]. This enables a lower consumption, in particular thanks to the elimination of the overflow losses due to the antechamber holes that have a strong throttling effect. The mixture formation experiences a significant improvement here thanks to the use of several injection holes and a higher injection pressure, creating smaller fuel droplets and already reducing particulate emissions [1]. Particles of all size categories are considerably reduced. The specific control of the injection start also has a major influence on particulate emissions [20].

**Figure 4:**
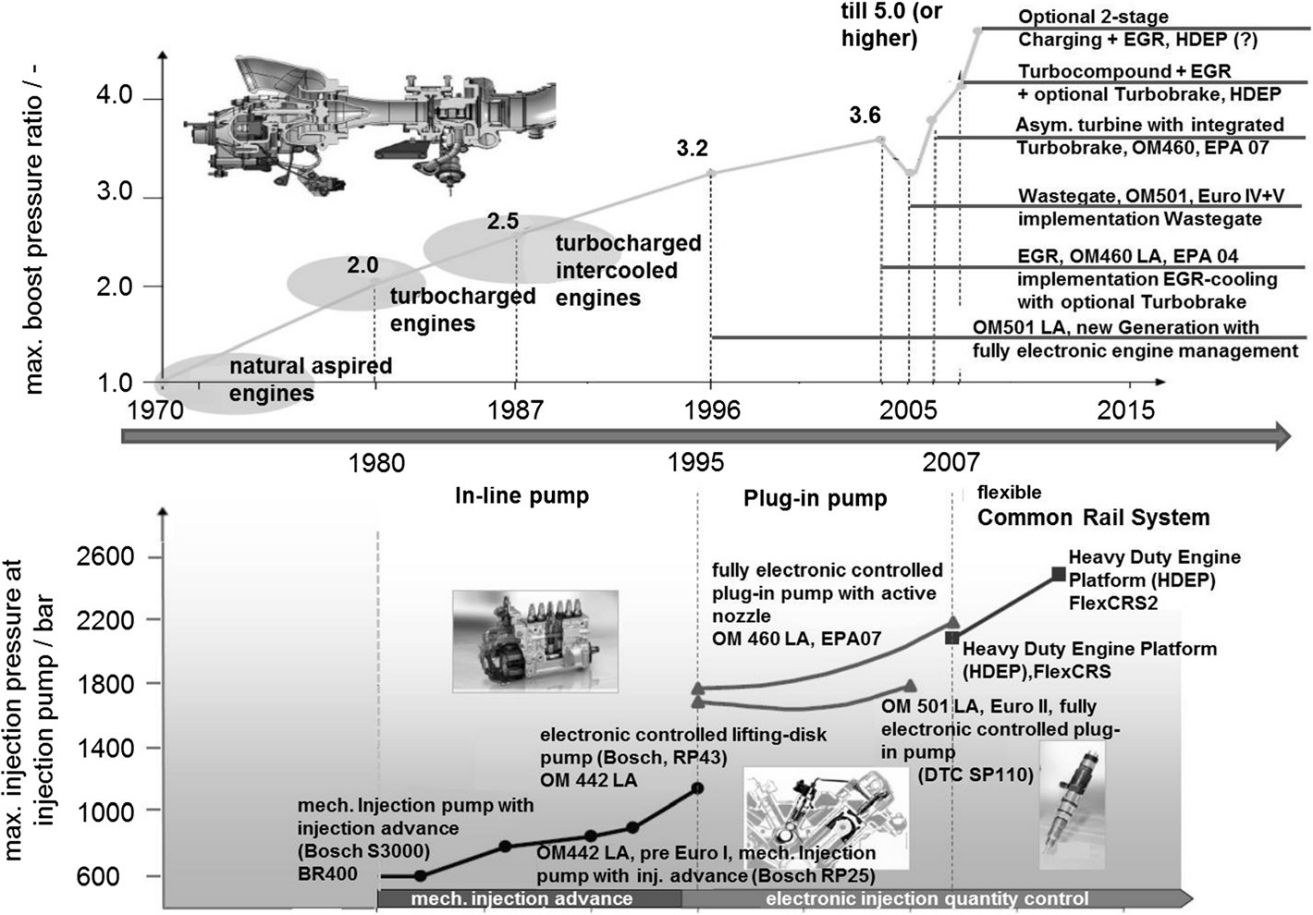
**Development of injection technologies, injection pressure and charging technology **[21]**.**

Until the 1990s, mechanically controlled injection methods were used for the most part, Figure 4 (below). They were replaced by electronically controlled systems, which permit higher pressures and flexible injection rate shaping. Common rail systems for example make it possible to separate the injection into several single fuel injections (pre-injection, main injection and post injection) with a variable start of injection and a variable injection duration [21]. The pressure curve and thus the noise development can therefore be influenced, or strategies for the specific support of the exhaust aftertreatment system can be implemented.

#### Exhaust gas recirculation (EGR)

**Figure 5:**
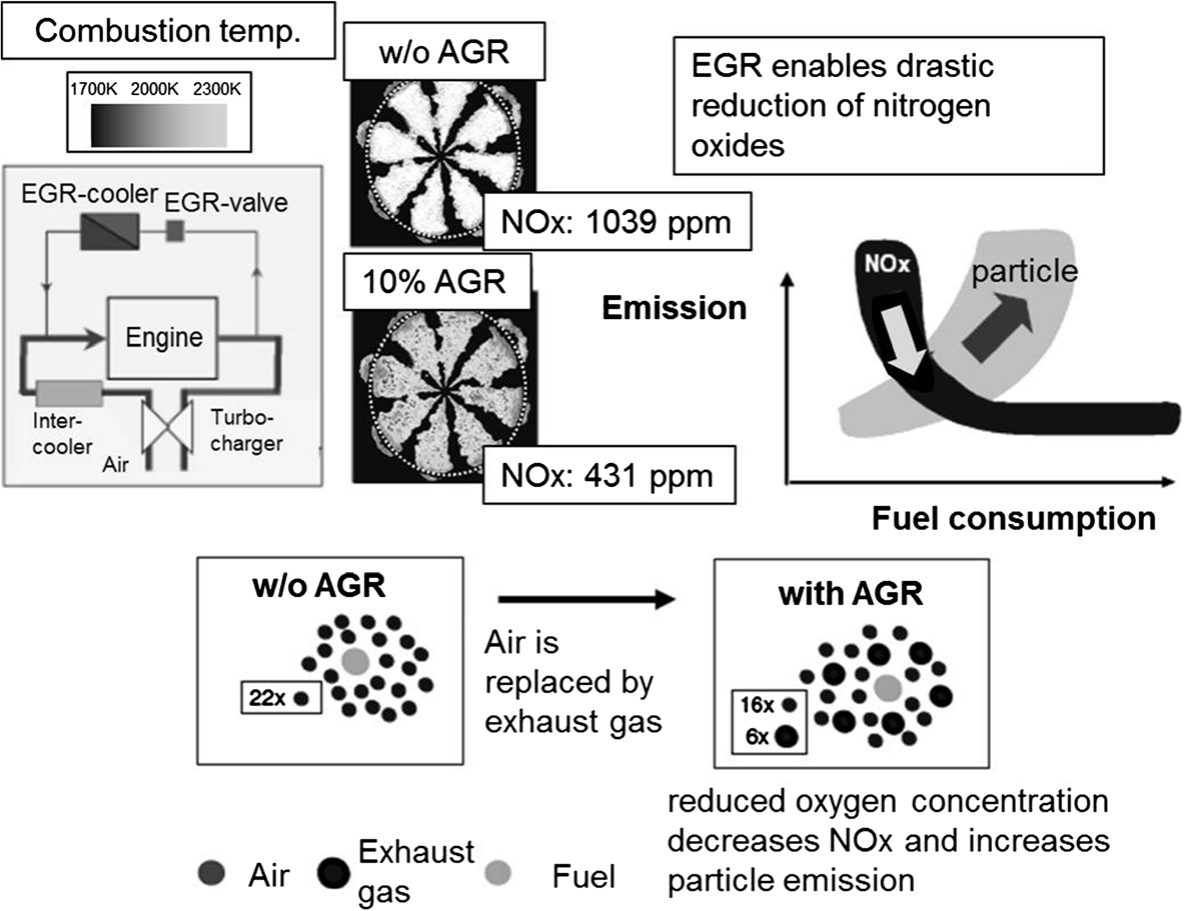
Overview exhaust gas recirculation.

Exhaust gas recirculation, where exhaust is added to fresh combustion air (Figure 5), has become an established method for reducing nitrogen oxides. A distinction can be made between in-engine and external exhaust gas recirculation. Both variants lower the peak combustion temperature. The increased specified heat capacity of the cylinder charge with EGR leads to a lower temperature increase when the same amount of heat is released than without EGR. Moreover, there is less oxygen in the combustion chamber, so that the nitrogen molecules encounter fewer reaction partners. Combustion is slower as well, which leads to a reduction in combustion temperature [22]. However, particulate emissions increase considerably, since soot formation becomes easier due to the reduced availability of oxygen, and soot oxidation becomes more difficult. Nitrogen oxide and soot emissions therefore behave in opposite directions when the engine operating parameters are adjusted; this is referred to as the particle/NO_x_ trade-off. The objective here is to displace the entire trade-off curve in order to reduce both particulate as well as the NO_x_ emissions.

In-engine EGR is implemented during the gas exchange process by returning exhaust to the combustion chamber when the cylinder is filled through simultaneously open intake and exhaust valves. In the external EGR, a distinction is made between high pressure and low pressure EGR. With high pressure EGR, which is currently the state of the art, exhaust is removed upstream of the turbocharger, cooled down, and admixed to the compressed fresh air downstream of the charge air cooler, and therefore at a high pressure level. The major advantage of external EGR compared to in-engine EGR is the possibility of cooling the recirculated exhaust, since the combustion temperature can be further reduced and the charge density is increased, allowing the engine to handle higher loads. This leads to an increase in the combustion air ratio, thus helping to oxidize soot and reduce soot emissions [1]. With low pressure EGR, exhaust is removed downstream of the particulate filter (DPF). The return into the combustion air takes place upstream of the compressor and therefore at a lower pressure level. The benefits are a good mixture in the compressor as well as efficient cooling in the charge air cooler, which improves the efficiency of the EGR. Therefore, less EGR is required for the same NO_x_ emission level. Additionally, the turbocharger is operated with better efficiency levels - compared to the high pressure EGR - due to the increased mass flow through compressor and turbine, making it possible to reduce gas cycle losses and thus fuel consumption. Due to the reduction of the EGR as well as the increased air supply, there is a lower particle formation and an improved particle after-oxidation. As a result, a reduction in particles can also be achieved by means of the low pressure EGR.

#### Charge movement

A targeted manipulation of the charge movement can improve the mixture formation and thus accelerate combustion. To generate the swirl flow, in other words air flow rotating around the cylinder axis [1], the geometry and arrangement of the inlet ports are the most important factors. Next to these elements, the piston geometry has an influence on the swirl flow due to the acceleration of the mixture mass in the recess (recess swirl) towards the end of the compression.

Depending on the load point, combustion system and injection system, more or less swirl can be beneficial for the particulate emissions which is why a tuned swirl level is important for low-emission combustion. Variable swirl can represent a further step for optimization. In passenger cars at low partial loads, a higher swirl level (partial closing of the filling port in a four-valve cylinder head) can lead to a better particle/NO_x_ trade-off, whereby consumption as well as CO emissions increase because of the deterioration in volumetric efficiency [1].

#### Supercharging

Nowadays, almost all diesel engines are supercharged, with exhaust turbocharging being the prevailing method. Here, energy is extracted from the exhaust flow and then used to drive a compressor [1]. During supercharging, the air needed for the combustion process is compressed, so that a higher air mass per working cycle is supplied to the cylinder. The injected fuel mass can therefore be increased, which in turn makes it possible to increase the engine output. Supercharging is therefore an important tool for enabling the so-called “Downsizing” – decreasing the engine without any changes to customer benefits (output, acceleration, etc.). There can also be other advantages in terms of efficiency and emissions. Figure 4 (above) provides an overview of the development of supercharging [21].

#### Compression ratio

A reduction of the compression has two positive effects from a thermodynamic perspective: It improves engine performance, since the same peak pressure is reached during full throttle with increased boost pressure and therefore more fuel can be injected with a constant combustion air ratio. Secondly, the temperature in the cylinder is reduced due to the compression during partial load due to the low cylinder pressure at constant boost pressure. This has a negative effect on the fuel’s ignition conditions, so that a better pre-mix of fuel and air is achieved. As a result, a significant improvement of the particle/NO_x_ trade-off can be realized. A reduced compression has a negative effect on engine behavior at cold ambient conditions with respect to combustion stability and emissions.

### External measures for reducing emissions

To reduce exhaust emissions, auxiliary exhaust aftertreatment systems are increasingly used for diesel engines [23-27]. The introduction of the corresponding technologies into mass produced passenger cars in Europe is shown in Figure 1. Diesel Oxidation Catalysts (DOC) and Diesel Particulate Filters (DPF) had already been mandated in order to comply with the Euro 5 limits in diesel passenger cars and were introduced across all of Europe. For the Euro 6 legislation, many applications will also require a system for nitrogen-oxide aftertreatment. In commercial vehicle engines, the Selective Catalytic Reduction (SCR technology) has become the norm for the most part in Europe since Euro IV. The reason for this is that, when a SCR system is used, the engine can be tuned in the direction of higher NO_x_ emissions and therefore generally in the direction of lower fuel consumption. Some manufacturers have also been using concepts with particulate reduction systems until Euro V. However, with the introduction of Euro VI, it is to be expected that all engines will use DOC, DPF, as well as SCR systems.

#### Diesel oxidation catalyst (DOC)

With the help of a DOC, HC and CO are oxidized at an adequate exhaust temperature to become CO_2_ and water. To do this, the rare metals platinum and palladium are applied to the catalyst support on a surface-enlarging wash coat. The temperature of the conversion of 50% is typically referred to as “Light-Off Temperature” and ranges between 150 and 350°C, depending on the catalyst type, emission composition, and catalytically effective coating. Typically, the CO Light-Off is achieved at lower temperatures than the HC Light-Off. If the Light-Off temperature has been exceeded, nearly all HC and CO emissions are converted. Thermal and chemical stress (e.g. sulfur poisoning) can reduce the function of the catalyst; the Light-Off moves in the direction of higher temperatures.

An oxidation of the soot particles is not possible in the DOC. Nevertheless, the DOC has important functions in conjunction with the exhaust aftertreatment of particulate emissions: Hydrocarbons (SOF) adhering to soot can be oxidized or cracked, thus reducing the particulate mass to a significant extent. In addition, the DOC forms NO_2_ due to the oxidation of nitric oxide, which can be used for passive soot filter regeneration thanks to the CRT® effect. The DOC can also be used as a catalytic burner that increases the exhaust temperature for active soot filter regeneration.

#### Particulate reduction systems

When using particulate reduction systems, a distinction must be made between the two operating modes soot accumulation and soot burn-off (regeneration). The systems for particulate reduction can be divided into “closed” wall-flow filters and “open” particle catalysts [28-30]. While closed systems force the exhaust to pass through a porous filter medium, a partial filtration of the particles takes place in open systems, whereby part of the exhaust flows through the filter without being filtered. The disadvantage of closed systems is a higher exhaust back pressure, which causes higher fuel consumption. Without any suitable countermeasures, it is possible that the DPF becomes clogged. Particle catalysts cannot clog up, since their filtration efficiency decreases with increasing loads. They are typically regenerated in a purely passive manner, so that no interaction with engine timing is required. This is why they are often used as retrofit systems.

**Figure 6:**
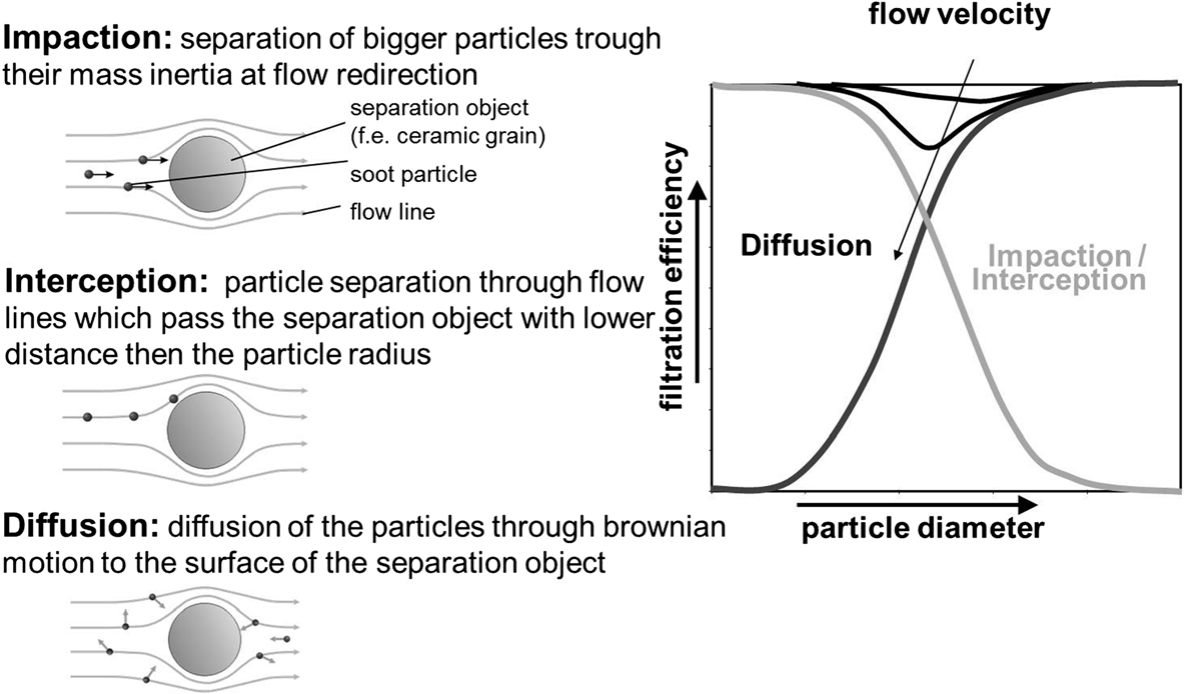
**Filtration mechanisms of diesel particulate filters**[6]**.**

A large filter surface is required in closed particulate filters in order to keep the soot layer thickness and thus the flow resistance low. To do this, the filter is designed as a honeycomb structure with alternately closed ports [1,31]. Asymmetric designs with larger intake and exhaust ports are also used to increase the storage capacity of the combustion residues (e.g. ashes) incurred during the course of the filter’s service life. Materials such as silicon carbide, aluminum titanate, and cordierite are used here in series production [32-39]. The filter efficiency depends on the pore size of the ceramics and the particle composition. Hydrocarbons with a high boiling point that are still present in gaseous form at filtering temperature and only adhere to the particles when they cool down and mix with the ambient air cannot be filtered out. Figure 6 shows the filtering mechanisms. Due to the overlapping filtration mechanisms, both large as well as small particles can be held back reliably, thus achieving a filtering efficiency of nearly 100% across the entire spectrum of sizes [6]. Since almost all emitted particles are smaller than the pores of the filter substrate, they are not caught in the filter due to their size but mostly by means of diffusion. Since the diffusion speed increases with decreasing particle size, smaller particles are actually separated the most effectively. With rising soot loads, there is a transition from deep filtration in the filter wall down to surface filtration. Both the soot layer stored in the pores as well as the soot cake on the filter wall itself act as a highly effective filtering medium. Due to the low deep filtering capacity of the ceramic honeycomb filter, the range of surface filtration is already being reached after a short load time. This is why a significant particle breakthrough in an intact filter can only be detected after the completion of an entire regeneration process in the startup phase of the load [39]. Particle catalysts can also reach particle filtration levels of considerately more than 50% [40], but these levels remain below those of wall-flow filters. For new systems, it is also possible to reach filtration efficiencies of more than 90% in combination with the filtration of nano-particles by means of electrostatic forces [41].

Due to the limited soot storage capacity, soot must be removed at regular intervals (typically every 500 – 1,000 km [42]) to prevent overload and thermal degradation of the material. The regeneration can take place continuously (passively via NO_2_ at T > 250°C and with an adequate NO_2_/soot mass ratio in the exhaust as well as a minimum soot quantity in the DPF [43]) or intermittently (thermally via O_2_, T > 600°C). The temperatures needed for thermal regeneration are often not reached during normal vehicle operation, which is why an additional temperature increase is required. The temperature can be increased by means of intake air throttling, adaptation of the EGR rate, modification to the injection management of pre-injection and main injection, introduction of early post injection in order to increase the exhaust temperature in the combustion chamber, as well as introduction of late post injection in order to increase the exhaust temperature via exothermic reactions on a DOC located downstream. Furthermore, fuel metering systems in the exhaust are used for exothermic reactions on a DOC that is located downstream, burner systems to increase the exhaust temperature, or electric heaters. Active regeneration measures increase fuel consumption, whereby late post injections can additionally lead to oil dilution. This is why additional measures are used in many cases to lower the regeneration temperature to levels below 600°C or to reduce the regeneration frequency. These include fuel additives to lower the soot ignition temperature, catalytic coating of the DPF, as well as acceleration of passive regeneration via NO_2_[44,45].

#### NO_x_ aftertreatment systems

Nitrogen oxides can be reduced in case of an excess of oxygen through Selective Catalytic Reduction (SCR), during which they are primarily converted into N_2_ and water using ammonia (NH_3_). SCR technology has been used for many years for flue gas nitrification in power plants. Under the prevailing stationary conditions and temperatures of between 300°C and 500°C, NO_x_ conversion rates of more than 90% are achieved. Optimizations have extended the temperature range down to 200°C, so that this technology has been used for several years now for passenger cars and commercial vehicles as well [43,46-48]. Today, commercial vehicles reach conversion rates of more than 90% in the test cycle; for passenger cars, the efficiencies are below those values due to the lower temperature level. Using gaseous ammonia in vehicles is questionable for safety reasons, which is why chemically bonded NH_3_, e.g. a safe urea/water solution is used that releases NH_3_ after being injected via catalytic hydrolysis, which is then used for NO_x_ reduction. The reactivity of an SCR catalyst can be increased by connecting a DOC ahead of the SCR. Due to the oxidation of NO into NO_2_, the kinetics of the catalytic process can be increased significantly through the intermediary formation of N_2_O_3_ in particular at T < 250°C [49].

For NO_x_ aftertreatment, it is also possible to use NO_x_ storage catalysts (NSC), which have been used for years in series production for lean engine operation gasoline engines and for some time now in diesel passenger cars [1,50-54]. The operating principle is based on the adsorption of the nitrogen oxides on storage elements (e.g. BaO) in the form of nitrate during lean engine operation (chemisorption). The first step here is the oxidation of NO into NO_2_ on the rare metal. The desorption and decomposition of the nitrate take place in short substoichiometric (rich) combustion phases with a reduced atmosphere in the exhaust. Oxygen deficiency and presence of unburnt hydrocarbons, CO, and hydrogen permit the reduction of the desorbed nitrogen oxides while forming N_2_, CO_2_, and water.

### Diesel fuel – type and grade

Table 2 shows an overview of different diesel fuels with their particulate emission properties. Advantages of GtL fuels with regard to particle formation were found by Kitano et al. [55]. The reduction of the soot emissions as well as the consumption at a constant NOx level by using pure HVO and/or blend of 70 percent by volume of EN 590 diesel with 30 percent by volume of HVO were verified by Aatola et al. [56]. Alternative, aromatics-free fuels such as HVO, GtL, or FAME therefore show a clear potential for reducing the relevant exhaust emissions. However, the statutory minimum for the admixture of biofuels only stipulates admixture rates of 3% up to a maximum of 10%. In these areas, there is only a low impact by the raised cetane number on the ignition delay and thus the burning characteristics [57].

**Table 2:** Overview of typical diesel fuel types

**Diesel fuel type**	**Typical methods**	**Typical properties**	**Influence on soot formation**
**Mineral-oil-based diesel fuel**	● Petroleum distillation	● Sulfur content	● Soot formation proportional to sulfur content
	● Mixture of approx. 200 hydrocarbons (alkanes, olefins, cycloalkans, aromatics)	● Aromatics content	
		● Boiling curve	
		● Low H/C ratio	● Soot formation increases in the following sequence: Alkanes → Cycloalkanes → Olefines → Aromatics
**Bio-fuel 1st generation**	● Transesterification of vegetable oils	● No sulfur content ● No aromatics	● O_2_ content causes low soot formation
	● FAME (Fatty Acid Methyl Ester)	● ~10% oxygen content	● Increased SOF portion in the particles
		● High boiling point	
**Bio-fuel 2nd generation & gas to liquid**	● Fischer-Tropsch process	● No or very low sulfur and aromatics content	● Low particle formation due to low sulfur and aromatics content and high H/C ratio
	-Biomass to liquid (BtL)		
	-Gas to liquid (GtL)	● No oxygen content	
	● Hydrogenated vegetable oil (HVO)	● High H/C ratio	
		● High cetane number	
		● Low spec. density	

## Effects of the technological progress on particulate emissions

**Figure 7:**
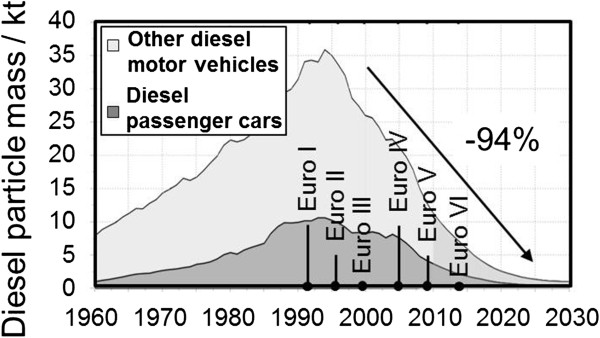
**Development of particulate emission through car traffic in germany **[58]**.**

The illustrated technological progress and the introduction of emissions standard have resulted in drastic emission reductions in recent decades. Figure 7 for instance forecasts a reduction of approximately 94% in the particulate mass emitted by commercial diesel vehicles relative to the maximum value. In addition to the mere emission levels, further modifications such as the particle size spectrum, morphology, and composition must also be analyzed. With regard to particle size distribution, only those sources of literature were considered as part of this study where it was possible to rule out a measurement of artifacts by means of correspondingly adjusted measurement technology.

### Effects of in-engine measures for emission reduction

**Figure 8:**
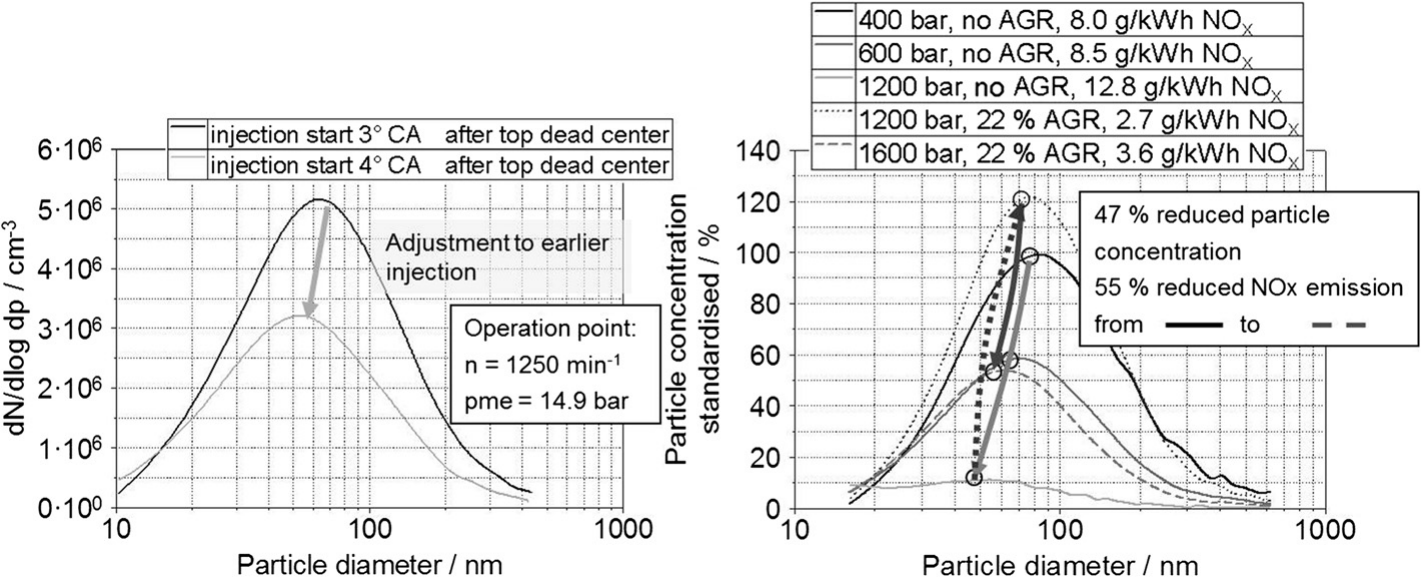
**Influence of injection and egr on particulate size distribution **[20,59]**.**

An increase in the injection pressure as well as an adjustment of the injection timing towards an “early” injection show a clear reduction in particle number emissions across the entire spectrum of sizes (Figure 8l.). For both measures, there is a slight shift of the maximum towards smaller particles here, but they are also emitted in smaller numbers. Both measures lead to a reduction in particulate mass emissions as well. Figure 8 (r.) also shows the influence of EGR and injection pressure on particle and NO_x_ emissions in a commercial vehicle engine. As a first step, the injection pressure was increased from 400 to 1,200 bar without EGR. While the NO_x_ emissions were increased, it was possible to considerably reduce the particulate mass emission (not shown) and the particle number across the entire spectrum of sizes due to the improved mixture formation. The introduction of the EGR led to a significant reduction in NOx emissions with a considerably higher particle number and particulate mass. By further increasing the injection pressure from 1,200 to 1,600 bar with a constant EGR, it was possible again to significantly reduce the particle number at only moderately increased NO_x_ emissions. When combining the previously described measures, it was possible to achieve a clear reduction in NO_x_ as well as particle number emissions across the entire spectrum of sizes with the introduction of EGR while simultaneously increasing the injection pressure.

It was possible to reach a similar reduction in the particle number emission by means of the transition from swirl chamber engines to direct injection engines, as well as under the boundary condition of injection systems improved across 3 generations, as found by Metz [60] in BMW passenger car diesel engines. In terms of the particle structure, no significant changes to the basic structure of the soot particles as well as their primary particle diameters were recognizable here. Further investigations of the influence of engine parameters and in particular the injection pressure on particulate emissions were illustrated by Mohr et al. [61]. A shift of the maximum in particle size distribution in the direction of smaller diameters at increased injection pressure was described here as well, whereby the overall particle number was significantly reduced and no emission increase in smaller particles was detected. Fiebig [62] conducted investigations with regard to the possibility of influencing the soot reactivity by varying engine parameters with the objective to represent improved soot properties for particulate filter regeneration. It was possible to show that, by varying in-engine parameters, soot reactivity – determined by combustion experiments in the thermogravimetric analysis – can be varied in a wide range.

### Effects of external measures on emission reduction

#### Diesel oxidation catalyst (DOC)

The DOC oxidizes the SOF adhering to the soot, which made it possible to substantially reduce the particulate mass. This was analyzed among other things by Krahl et al. [63] on a commercial vehicle engine with different fuels (diesel as per DIN EN 590, RME, B5Ult, V-Power) and confirmed for all fuels. Both fuel-based as well as oil-based SOFs are reduced in the process.

**Figure 9:**
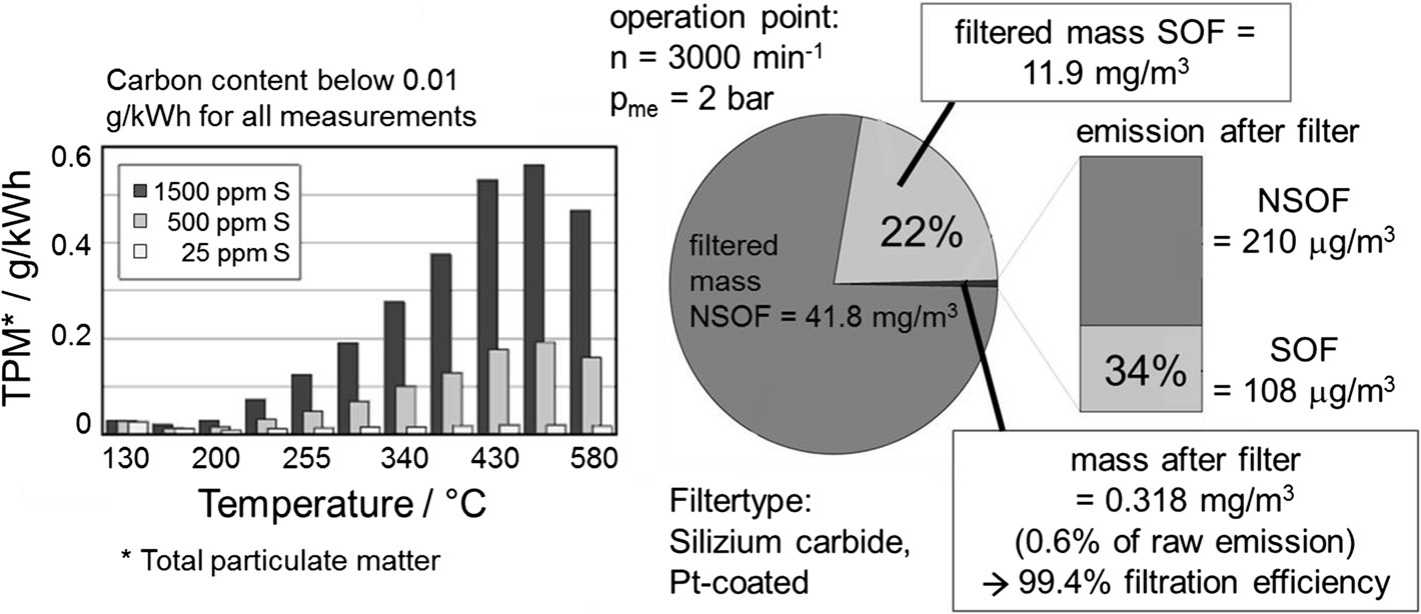
**Particle formation through sulfates on DOC **[64]** (left) and influence of DPF on particle composition (right).**

Using a platinum-based DOC the significance of low-sulfur fuel for particulate emissions as a function of the temperature for 3 different sulfur contents is shown in Figure 9 (l.). With a sulfur content of 500 ppm and even more pronounced with a sulfur content of 1500 ppm in the fuel, we can see a significant increase in particulate emissions – due to the sulfate formation via the DOC [64].

#### Particulate reduction systems

**Figure 10:**
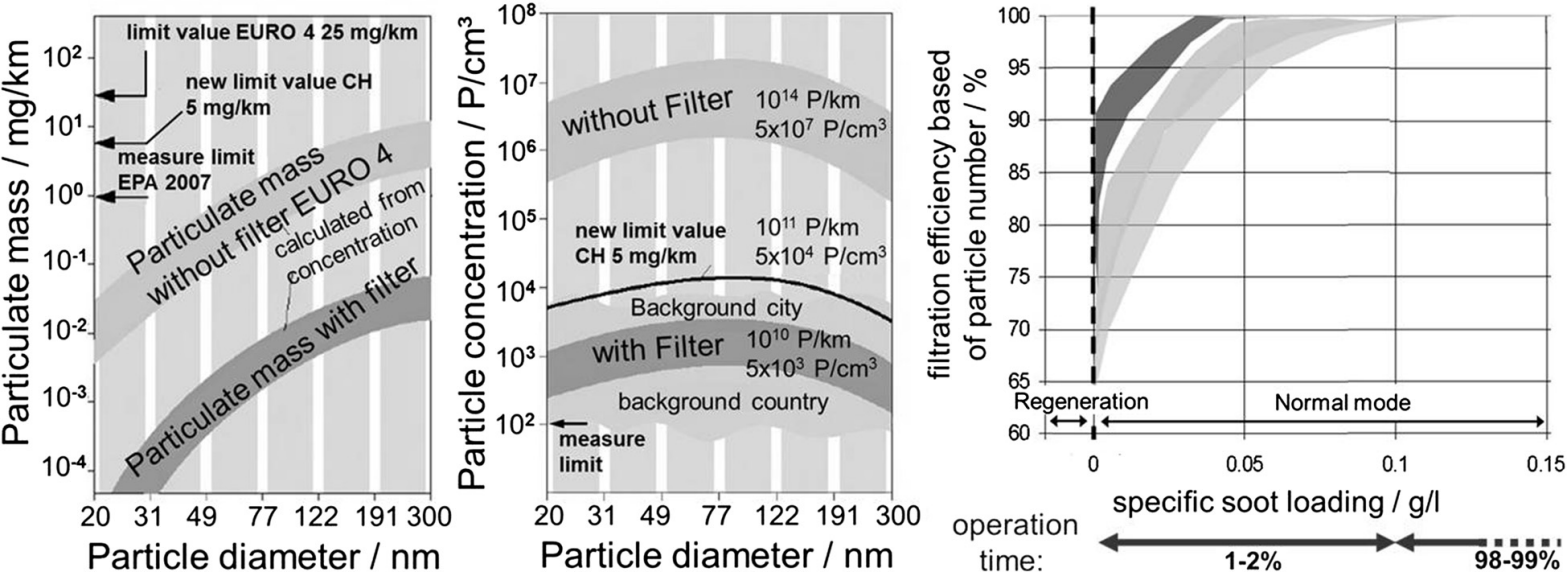
**Particle reduction through DPF **[68]** (left, mid) and particle number reduction based on filter load (right).**

For 2 DPF substrates used in series production, Zikoridse [65] shows that, by using a closed particulate filter, the emitted particle number can be reduced by several orders of magnitude across the entire spectrum of sizes and that the particle number concentration downstream of the DPF is within the range of the ambient air concentration. The same is shown in Figure 10 (left, center) for a diesel passenger car with DPF. Here too, both the particle number as well as the particulate mass is reduced by several orders of magnitude and the particle number concentration is within the range of the background level. With ADAC [66], it was possible to show for different legal test cycles as well as for further driving conditions that the particle number for a vehicle with DPF is below that of a vehicle without DPF by several orders of magnitude regardless of the cycle. At a constant speed of 80 km/h, a vehicle with DPF on average emits an approximately 10,000 times lower particle number. The particle number concentration is also within the range of the background level here. Schmidt [67] shows that the particulate mass is reduced by at least 2 orders of magnitudes with a closed DPF on a commercial vehicle engine.

The filtration efficiency of particulate filter largely depends on the soot load of the filter and generally rises with increasing soot load. This is shown in Figure 10 (r.) for different filter substrates. To start with, the filters were completely emptied in regeneration mode and loaded in normal mode, whereby the particle number was measured continuously and used to calculate the filtration efficiency for the particle number. The concentration-related filter efficiency in unloaded condition was between 70 and 90% and quickly rose with increasing load to close to 100%. The time until the maximum efficiency is reached was only 1 to 2% of the regeneration interval, so that a high efficiency was achieved across the entire operating time. The fast increase in filtration efficiency with increasing filter load was also made visible by Kirchner et al. [69] in the NEDC. The influence of the DPF on the particle number emission in commercial vehicle engines was also illustrated by Khalek et al. [70], whereby the influence of active DPF regeneration was also shown here. The particle number with engine technology from model year 2004 (without DOC and DPF) was compared to technology from 2007 with actively regenerated DPF in different test cycles. It is evident that, even with active regeneration, the reduction in particle number for the technology from 2007 is at roughly 90% and without regeneration, it reaches more than 99%. A detailed analysis of the particle composition in a passenger car diesel engine upstream of and downstream of the DPF for 2 stationary engine operating points showed that high mass-related filtration efficiencies were achieved with a wall-flow filter for both the non-soluble fraction (primarily soot) as well as for the soluble fraction (Figure 9 r.). The filtration efficiency for the solid content was higher here than for the soluble portion (which depends on the operating point).

The particulate mass reduction on the particle catalyst for a commercial vehicle engine was shown by Emitec [71] for the ESC and ETC. test cycle. A result of 70% resp. 64% was achieved and the values were below the Euro IV particulate limits. The filtration efficiency can be influenced by means of the design of the system among other things and increased beyond the values shown by Emitec [71], but this typically results in a conflict between the filtration efficiency and the exhaust back pressure of the filter [8]. Scheeder et al. [72] also showed on a commercial vehicle engine that, in addition to reducing the particulate mass by using a particle catalyst, the emitted particle number can be clearly reduced [8]. In this example, there was a nearly constant filtration efficiency of approximately 70% across the entire spectrum of sizes. Therefore, partial flow systems also represent an effective measure for reducing particulate mass and particle number.

Results by Kirchner et al. [69,73] indicated which variation must be observed when analyzing the particle number emission measurement. Based on the results of the particle number emission measurement in the NEDC of new, conditioned vehicles, it appears that there can be wide variations for different vehicles of the same type and model and that, even for repeated measurements performed on the same vehicle, differences in the range of roughly 100% must be taken into account. The comparison of 3 different methods for particulate measurement purposes (2 methods for particle number emission measurement, one method for particulate mass) showed that the differences are for the most part due to the variations in vehicle emissions and not due to inaccuracies in the measurement technology. The repeatability and reproducibility of the particulate mass and particle number emission measurement in a Euro IV commercial vehicle engine was also shown in detail by Andersson et al. [74] for different cycles and dilution systems. It is also apparent here that the variabilities of engine and/or particulate filter have a major influence on the particle number emissions.

#### NO_x_ aftertreatment systems

**Figure 11:**
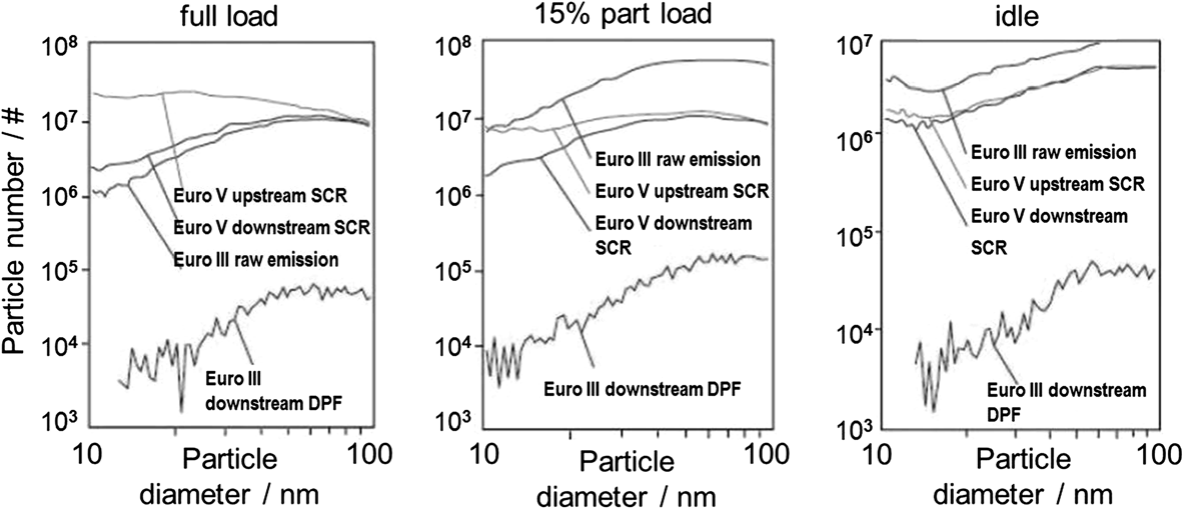
**Influence of SCR on particulate number (commercial vehicle) **[75]**.**

The SCR method operates with a urea/water solution that is introduced as an additional operating fluid to the exhaust. The influence on particulate emissions is illustrated in Figure 11 based on investigations in a Euro V commercial vehicle engine with SCR in comparison to a Euro III concept with DPF. Upstream of the SCR catalyst, we can see a clear increase in solid micro-particles in the Euro V engine that rises with increasing load and that raises the particle concentration of < 30 nm by more than one order of magnitude at full throttle. Possible causes for this are the formation of sulfates, mineral particles, or reaction products from the reduction agent [75]. However, the particle number is reduced again by the SCR catalyst to a level that is comparable to the Euro III without DPF (full throttle) resp. below that level (partial load and idling). It should be noted in that context that a DPF will also be used in addition for a future Euro VI concept, resulting in a clear further reduction in particulate emissions down to or below the level of the Euro III engine with DPF. Particulate emissions will therefore not be increased due to the SCR system.

Mohr [76] analyzed the correlation between the solid particle number and the soot mass for a commercial vehicle engine with SCR system for different driving cycles and measurements with and without SCR catalyst as well as with and without urea injection. Particle number and soot concentration show a good correlation when using a comparable engine load and combustion strategy. Using the SCR technology, it was not possible to find any negative effects on particle number emissions, since there was no tendency of a correlation shift in the direction of an increased particle number emission in any of the analyzed cycles.

To regenerate the stored nitrogen oxides, intermittent short substoichiometric (rich) engine operation is required in the NSC, which differs fundamentally from lean diesel operation. Investigations in 4 engine operating points performed by Pischinger et al. [77] showed that the rich engine operation can differ significantly from lean engine operation with regard to particulate mass and composition depending on the operating point. During operation at low loads of 1,395 rpm/1.9 bar brake mean effective pressure, the overall particulate emissions during rich engine operation showed a clear increase, while the particulate mass was mainly due to adhering SOFs from post injection. By contrast, at the operating point of 1,500 rpm/3.2 bar brake mean effective pressure, the overall particulate emissions mainly rose caused by a higher solids content. At 3,000 rpm/2 bar, the particulate emission during rich engine operation was even decreasing. In contrast to lean engine operation, we see a higher portion of insoluble organic pyrolyzate, which decomposes at temperatures of 500 to 600°C, for most operating points in terms of the overall particulate mass during rich engine operation. It is worth noting here that the effects of rich engine operation on particulate emission and composition largely depends on the strategy selected for realization of the rich engine operation and that it is still subject to significant changes through the catalyst system. If we also take a look here at the particle size distribution by comparing lean and rich engine operation, we can see that a higher soot emission during rich engine operation is not due to an increase in particle number, but based on a shift in distribution towards larger diameters.

### Effects of fuel grade and type

Direct importance is attached to the reduction of the fuel’s sulfur content in terms of particulate emissions of diesel engines, since - in particular when using a DOC - the sulfuric acid generated from the fuel’s sulfur, contributes to the particulate mass because of the legally required analysis method, and it is therefore not possible to achieve stringent PM limits with high sulfur contents in the fuel (cf. Figure 9l.). Walker [78] illustrates this for a commercial vehicle engine with a particulate emission level that is relevant for emission standard Euro IV resp. Euro V. With high sulfur contents, sulfate generation contributed to the particulate mass to an extent that the limits are exceeded. This portion was also not reduced with a DPF. It must be noted that it was possible to achieve an immediate improvement in (particulate) emissions for all vehicles on the market with the introduction of nearly sulfur-free fuels in Europe – in particular for older vehicles as well. In this context, it is also worth mentioning the efforts towards a global harmonization of fuel grades [79].

Different fuels lead to soot emissions with a different level of graphitization, which has an effect on morphologic properties as well as exhaust aftertreatment. A higher bio-diesel portion generally leads to slightly elevated nitrogen oxide emissions with simultaneously clearly reduced particulate mass emissions, which can be explained with the higher oxygen portion in the fuel among other things. This was confirmed by the results from Czerwinski et al. [80] for rape methyl ester (RME) and rape seed oil fuel (ROR), in particular at higher engine loads. The particle number concentration also did not show an increase for RME; it even decreased substantially for ROR. The spec. surface was clearly reduced for both biofuels in comparison to the other fuels. These results were confirmed by data from Munack et al. [81] and Krahl et al. [63] and information was added regarding the particle size distribution and structure. To do this, investigations were conducted in a Euro III commercial vehicle by performing an ESC test with 4 different fuels (conventional diesel fuel, RME, Aral Ultimate diesel with 5% RME admixture, and Shell V-Power diesel). In comparison to all other fuels, RME had the lowest particulate emissions both upstream of and downstream of the DOC, which can be attributed for the most part to a lower solid fraction. In the particle composition investigations, it was possible to verify a higher portion of unburnt fuel on the particles for RME. However, these SOFs can be reduced through a DOC [81]. Particle number and masses showed the lowest values for RME; when comparing the 3 remaining fuels to each other, they showed a similar emissions behavior regarding the particle number. Additional investigations with SEM in terms of the particle structure did not show any dependence on the fuel used [81]. Trapel & Roth [82] also found a reduction in the particle number with an increasing portion of FAME (bio-fuel). In the process, it increased for small particles with a rising FAME portion. The influence of fuel contents in bio-fuel with a high boiling point on the measurement cannot be ruled out here. It was not possible to find any influence of bio-diesel on the particle structure. The results show comparable primary particles and agglomerate sizes in a direct comparison between soot from FAME and soot from diesel. Investigations shown by Boehman et al. [83] on the structure of soot particles for fuels with a 20% bio-diesel portion indicated a significantly less organized (amorphous) structure in comparison to conventional diesel fuel. Possible causes mentioned are a different time/temperature history in the combustion chamber as well as the different fuel composition and thus different fuel decomposition and soot generation processes.

### Development of particulate emissions in the overall system

**Figure 12:**
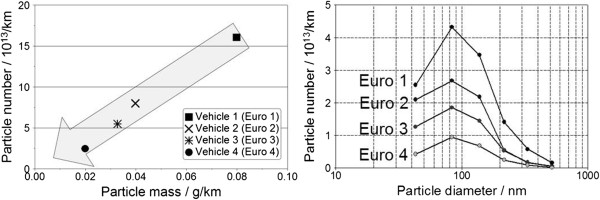
**Development of raw particle emission using the example of a diesel passenger car, VW Golf, NEDC **[86]**.**

The direct comparison of engines and/or vehicle across several emission standards is designed to show the effects of the modified engine and exhaust aftertreatment technologies on particulate emissions. Schwizer & Löhrer [84] performed a comparison of particulate emissions for different vehicle speeds for one vehicle each with Euro 3 (without DPF), Euro 4 (with DPF), and Euro 5 (with DPF). In the transition from Euro 3 to Euro 4/5, it was possible to achieve a clear reduction in particulate mass emissions across the entire vehicle speed range by using the DPF. Figure 12 uses the example of the VW Golf to show the development of the particulate mass and particle number emissions as well as the particle size distribution in terms of untreated raw emissions from emission standard Euro 1 down to Euro 4. A further possible reduction of the particle number starting from Euro 4 by using a DPF had not been taken into consideration in this comparison. The illustrations clearly show that it was possible to substantially reduce the particulate mass by means of a combination of engine measures and - in almost direct correlation - the particle number emission. The particle number could be reduced here evenly across the entire spectrum of sizes. ACEA [85] carried out investigations with regard to the particulate mass and the particle number using a wide variety of engine technologies from different manufacturers. Both for passenger car as well as for commercial vehicle engines, it was possible to find a decrease in particle number in correlation to the decrease in particulate mass within a range of variation. Schindler [86] was also not able to find a trend in the direction of smaller particles for the advanced diesel process in comparison to older engines with regard to the particle size distribution. It is therefore not possible to substantiate a negative influence on the particle number emission by the technological measures for emission reduction.

Kirchner et al. [69,73] analyzed the correlation between particle resp. soot mass and particle number by conducting measurements on different vehicles without and with DPF. We can see that there is a roughly linear correlation between particulate mass and particle number emissions for vehicles without DPF, which is approximately 2 * 10^12^ particles per mg. The correlation can also be transferred to vehicles with DPF, since soot is the major component when it comes to particles in advanced diesel engines that are equipped with a DOC and due to the fact that DPFs have a consistently high filtration efficiency across the whole particle spectrum. For the Euro 6 limit, this correlation results in a soot mass of only 0.3 mg/km for a particle number of 6 * 10^11^ particles/km, which is below the limit by a factor of more than 10. Based on this observation, the particle number will be the decisive factor for passenger cars in the future in the Euro 6 legislation.

## Effects of the technological progress on other emissions

The traffic-related nitrogen oxide emissions in the Federal Republic of Germany have fallen sharply since the introduction of the emission limitation for road traffic in 1960 up until today and a further reduction by a total of 86% in maximum emissions is expected in the future as well [58]. Ifeu [87] provided an overview of the development of emissions by commercial vehicles in the Federal Republic of Germany since 1980 as well as their percentage of total emissions for the year 1994. The volume of commercial vehicles rose by 50% during this period of time and included approximately 45 million vehicles in 1999. In spite of the growing numbers, there has been a sharp decline in pollutants such as HC and CO by 94% resp. 90% emitted in road traffic since Euro 1. With 50%, CO_2_ is the largest contributor to today’s anthropogenic greenhouse effect. It develops whenever any type of fossil fuel is burned and therefore represents the main greenhouse gas created during engine combustion. Traffic is currently producing 19% of CO_2_ emissions in Germany. The CO_2_ emissions of an internal combustion engine resp. of a vehicle are directly proportional to its consumption. During the complete combustion of one liter of diesel fuel, approx. 2,650 g of CO_2_ are generated depending on the fuel composition. Therefore, the fuel consumption in l/100 km must be multiplied by a factor of 26.5 to obtain the CO_2_ emissions in g/km [1].

The California Air Resources Board [88] analyzed the influence of different exhaust aftertreatment systems (uncoated DPF, coated DPF, combined DPF and SCR concepts) on PAH (polycyclic aromatic hydrocarbons) as well as nitro PAH emissions. A reduction of the entire PAH emissions of over 90% was measured for the analyzed exhaust aftertreatment systems. The reduction of the particulate PAH was above 95% here and was independent from the catalytic coating, whereas the reduction of the gaseous PAH was influenced by the catalytic coating as well as the exhaust temperature. We can also see that emissions of 1-nitropyrene (the dominant nitro PAH) without exhaust aftertreatment were higher by several orders of magnitudes in comparison to engines both with coated as well as uncoated DPF. Uncoated DPFs showed higher emissions of 3-nitrophenanthrene here in comparison to the basis. The two analyzed SCR systems did not lead to an increase in nitro PAH development [88]. It can also be noted that there was a positive effect from the DPF for these emissions. In the ACES study, the Health Effects Institute [89] also pointed out the possibility of the generation of nitrogenous compounds, including nitro PAH among other things, in combination with the SCR technology.

Based on a comparison of commercial vehicle engine technologies of the 2004 model year with those of the 2007 model year (with EGR and DPF), Khalek et al. [70] showed a reduction of non-limited emissions for advanced engine technologies in a 16-hour test cycle.

## Summary and look ahead

Diesel engines in both passenger cars as well as commercial vehicles have undergone a rapid development in the past 30 years towards more efficiency, environmental protection and comfort. The technical developments responsible for the drastic emission reductions include all kinds of aspects ranging from the fuel to the engine’s combustion system down to the exhaust aftertreatment. The development of the engine control has also been a major influencing factor. While engine control used to be purely mechanical in the past, electronic control units are now used in most cases. Depending on the operating point, this permits the precise control of the engine components and the combustion cycle, something that cannot be done with mechanical means. These significant changes over the past decades must be considered when evaluating particulate emissions in Euro 1 engines in comparison to advanced Euro 6 engines.

Nowadays, in-engine measures for optimizing combustion management permit the minimization of particles and nitrogen oxides at reduced consumption and higher output. Cooled exhaust gas recirculation effectively reduces NO_x_ emissions. Improved injection systems with greatly increased injection pressure and multiple injections permit a better mixture formation and a reduction of the combustion temperature, thus reducing NO_x_ and particles. Using adjusted boosting systems with charge air cooling, it is possible to implement increased boost pressures. They lead to a higher cylinder charge, resulting in a lower combustion temperature with lower NO_x_ emissions as well as a leaner combustion air ratio and therefore lower particulate emissions. By optimizing the combustion systems with regard to the charge movement in the combustion chamber as well as the geometry of the combustion chamber, the mixture formation can be further improved. Overall, particulate emissions can be reduced substantially with the described in-engine measures without causing a negative effect on the particle size distribution towards smaller particles.

Thanks to the introduction and improvement of exhaust aftertreatment systems, the pollutant concentration was also reduced considerably. The diesel oxidation catalyst (DOC) for example reduces the concentration of hydrocarbon and carbon monoxide emissions by nearly 100% after it has reached its operating temperature. Additionally, the DOC reduces the hydrocarbons adhering to the soot particles, while the portion of elemental carbon remains almost unchanged. The development of sulfates at the DOC no longer poses a significant problem for today’s mostly sulfur-free fuels in Europe. The remaining particles are effectively collected in the diesel particulate filter (DPF), whose filtering efficiency with closed design is near 100%, regardless of the particle size or operating mode of the engine. In addition to the closed particle filtering systems, so-called particle catalysts are also used in some applications, which have lower filtration efficiencies than a closed filter because of their principle. However, the particle number across the entire spectrum of sizes will be reduced here as well. SCR catalysts (Selective Catalytic Reduction) and NO_x_ storage catalysts are used for NO_x_ reduction downstream of the engine.

The fuel grade has also improved over the past few decades. The permissible sulfur content in the period from 1965 to 2005 for example has dropped from 1% to 0.005%, which lead to an immediate reduction in particulate emissions. In addition to mineral oil-based diesel fuels, 1st generation biofuels (FAME, RME, hydrogenated vegetable oil) as well as gas-to-liquid are increasingly used. These generally have a positive influence on particulate emissions and do not lead to an increase in particle number emissions.

In conclusion, the particulate emissions of advanced diesel engines can be drastically reduced in terms of the particulate mass and the particle number by using closed particulate filters. In-engine measures also lead to a clear reduction in particulate emissions. When measuring particle number and mass, we can see a clear correlation. Reduced particle mass emission is always associated with a reduction in particle number. Statements claiming that advanced engines are emitting a particular high amount of small particles were proven incorrect since they are based on measurement errors. There is no significant increase in small particles in the range of < 30 nm at the engine outlet because of advanced engine concepts. Particulate filters that were universally introduced for passenger cars with emission standard Euro 5 and became the state-of-the-art with Euro VI in commercial vehicles as well, are filtering particles in the entire operating range of the engine across the entire particle size range with high efficiency, which can be explained by the separation principle in the filter.

With the introduction of Euro 6 for passenger cars, it was possible to further reduce the permissible emissions especially for nitrogen oxides; with the introduction of Euro VI, both the particulate as well as the NO_x_ emissions were further reduced drastically for commercial vehicles. With Euro 5b for passenger cars and Euro VI for commercial vehicles, a particle number limit has been introduced additionally that drastically increased the requirements even further. Since the introduction of the exhaust emission standards in Europe, all pollutant components have already been reduced by more than 90%. Additionally, increasingly stringent measures are being introduced for monitoring emissions in real-life driving operation over the life time of the vehicle. Further steps in the legislation for reducing the emission limits are to be expected in the future. In addition, the focus will increasingly be on CO_2_ emissions.

### Annotation

A similar review article in the German language has been published by the Zentralblatt für Arbeitsmedizin, Arbeitsschutz und Ergonomie (M. Fiebig, A. Wiartalla, A. Kolbeck, S. Kiesow: Wechselwirkungen zwischen Dieselmotortechnik und -emissionen mit dem Schwerpunkt auf Partikeln. Zbl Arbeitsmed 63 (2013) 4–22).

## Abbreviations

°CA: Crank angle; B5Ult: Aral ultimate diesel with 5% RME addition; B20: Diesel fuel with addition of 20% biodiesel; BaO: Barium oxide; BtL: Biomass to liquid; C: Atomic carbon; CO: Carbon monoxide; CO_2_: Carbon dioxide; CRT®: Continuously regenerating trap; DI: Direct injection; DOC: Diesel oxidation catalyst; DPF: Diesel particulate filter; EC: Elemental carbon; EGR: Exhaust gas recirculation; ELR: European load response test; ESC: European stationary cycle; ETCa: European transient cycle; FAME: Fatty acid methyl ester; GtL: Gas to liquid; H: Atomic hydrogen; H_2_O: Water; HC: Hydrocarbons; HDEP: Heavy duty engine platform; HVO: Hydrated vegetable oil; IDI: Indirect diesel injection; KBA: Kraftfahrtbundesamt (Federal Motor Transport Authority); N_2_: Nitrogen; N_2_O: Dinitrogen monoxide; N_2_O_3_: Dinitrogen trioxide; NEDC: New european driving cycle; NH_3_: Ammonia; NO: Nitrogen monoxide; NO_2_: Nitrogen dioxide; NO_x_: Nitrogen oxides; NSF: Non-soluable fraction; NSC: NOx storage catalyst; NSOF: Non-soluable organic fraction; NTE range: Not-to-exceed range; O_2_: Oxygen; OBD: On board diagnosis; PAH: Polycyclic aromatic hydrocarbons; PM: Particulate matter; PN: Particulate number; ppm: Parts per million; RME: Rape methyl ester; ROR: Rape seed oil fuel; SCR: Selective catalytic reduction; SEM: Scanning electron microscope; SOF: Soluble organic fraction; T: Temperature; TPM: Total particulate matter; V-Power: Shell V-Power Diesel; WHSC: World harmonized stationary cycle; WHTC: World harmonized transient cycle.

## Competing interests

The authors declare that they have no competing interests.

## Authors’ contribution

MF and AW conducted the literature review and drafted the manuscript. BH coordinated the drafting of the manuscript. SK drafted the figures. All authors read and approved the final manuscript.
